# Understanding Sorption Mechanisms Directly from Isotherms

**DOI:** 10.1021/acs.langmuir.3c00256

**Published:** 2023-04-18

**Authors:** Seishi Shimizu, Nobuyuki Matubayasi

**Affiliations:** † York Structural Biology Laboratory, Department of Chemistry, University of York, Heslington, York YO10 5DD, United Kingdom; ‡ Division of Chemical Engineering, Graduate School of Engineering Science, Osaka University, Toyonaka, Osaka 560-8531, Japan

## Abstract

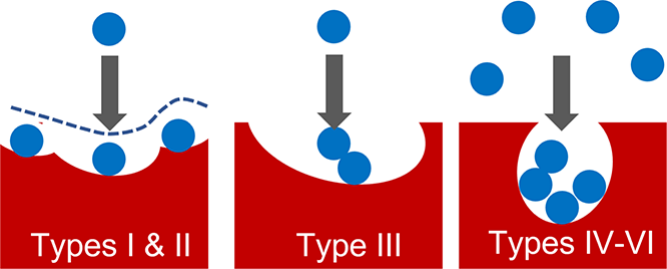

Currently, more than 100 isotherm models coexist for the six IUPAC isotherm types. However, no mechanistic insights can be reached when several models, each claiming a different mechanism, fit an experimental isotherm equally well. More frequently, popular isotherm models [such as the site-specific models like Langmuir, Brunauer–Emmett–Teller (BET), and Guggenheim–Anderson–de Boer (GAB)] have been applied to real and complex systems that break their basic assumptions. To overcome such conundrums, we establish a universal approach to model all isotherm types, attributing the difference to the sorbate–sorbate and sorbate–surface interactions in a systematic manner. We have generalized the language of the traditional sorption models (such as the monolayer capacity and the BET constant) to the model-free concepts of partitioning and association coefficients that can be applied across the isotherm types. Through such a generalization, the apparent contradictions, caused by applying the site-specific models alongside with cross-sectional area of sorbates for the purpose of surface area determination, can be eliminated straightforwardly.

## Introduction

Can the mechanism of sorption be revealed by analyzing experimental isotherms? Various isotherm models have been developed to answer this question.^1,2^ However, the difficulty comes from the diverse functional shapes that isotherms exhibit;^3−6^ the six types of isotherms, according to the IUPAC classification,^3−6^ have been analyzed using more than 100 isotherm models proposed so far.^7−13^ Such a practice, unfortunately, has made this simple question even more complicated for the following reasons. First, multiple isotherm models, each assuming a different adsorption mechanism (or even none), are capable of fitting the same data with comparable *R*^2^ values.^14−17^ Second, the most popular isotherm models, such as the Brunauer–Emmett–Teller (BET)^18,19^ and Guggenheim–Anderson–de Boer (GAB),^20−22^ have been applied routinely to real systems (e.g., food samples^23−25^ and construction materials^26^) that do not satisfy their original assumptions (i.e., site-specific, planar, layer-by-layer adsorption). The conundrum has led to the pessimism that “isotherm’s shape alone does not contain enough information to uniquely identify and quantify the underlying sorption mechanisms”.^14^

In response to this pessimism,^14^ we have recently shown that the underlying sorption mechanism can indeed be identified from an isotherm;^27−30^ sorbate–sorbate and sorbate–interface interactions can be quantified from isotherm’s shape alone with the help of statistical thermodynamics.^27−30^ (Our elaboration below employs the statistical thermodynamic notation and the theoretical foundation summarized in Appendix A.^27,28,30^) Our tools for achieving this are the statistical thermodynamic quantities for characterizing solution-phase interactions^31−35^ that have been generalized for interfaces. The sorbate–sorbate interaction is quantified via the sorbate excess number, *N*_22_, which is the difference in the number of sorbate molecules around a specific sorbate molecule (probe) from the one without the probe. *N*_22_ can be evaluated directly from how the amount of sorption ⟨*n*_2_⟩ depends on sorbate activity *a*_2_.^27,28,30^ From *N*_22_, the sorbate–sorbate Kirkwood–Buff integral (KBI) can also be evaluated when normalized by vapor concentration. The sorbate–interface interaction is quantified via the sorbate–surface KBI, which is the surface excess of the sorbate normalized by its vapor concentration. Through the quantification of sorbate–surface and sorbate–sorbate interactions from an isotherm in a model-independent manner, the underlying sorption mechanism can indeed be revealed from an experimental isotherm.^27−30^

This leaves the two remaining issues to be identified in the opening paragraph, i.e., (a) complications arising from the multiplicity of isotherm models and (b) the application of popular isotherm models beyond their original assumptions. Our goal is to resolve these difficulties by replacing the highly idealized isotherm models with the universality and model-free nature of our statistical thermodynamic theory. As a start, we have shown recently that the isotherm equations generated from our theory are capable of modeling IUPAC Types I, II, and IV–VI,^28,30,36,37^ in contrast to the traditional isotherm models that involved a different presumed mechanism (or more) for each isotherm type. This has been achieved in our recent papers^27−30^ based on a key relationship between an isotherm and the underlying KBIs. (see the Theory section for details). Incorporating the sorbate pair and triplet contributions to the sorbate–sorbate KBI, as well as the sorbate–surface KBI at the dilute limit, has led to the general statistical thermodynamic isotherm. This isotherm, referred to as the “ABC isotherm”, contains the Langmuir, BET, and GAB models as its special cases.^28,30^ The ABC isotherm was successful in fitting experimental data (such as the water and nitrogen adsorption on a Portland cement sample and nitrogen and argon adsorption on Zeolite X13^30^), quantifying the underlying interactions, and clarifying the insights into the underlying sorption mechanism.^28,30^ Most importantly, the ABC isotherm has provided a long-sought explanation as to why the Langmuir, BET, and GAB models can be applied successfully to model the systems (e.g., food and cement) that break the fundamental assumptions of their site-specific and layer-by-layer adsorption mechanisms.^27,28,30^ The concepts like the sorbate–surface, sorbate pair, and sorbate triplet interactions are universal and model-free, and are sufficient to account for the Type I and II behaviors.^27,28,30^

However, we must bear in mind how much of our thinking has been shaped by the isotherm models, especially the Langmuir, BET, and GAB. Hence, our quest for a universal sorption theory necessitates a full elucidation of what the commonly used model parameters (such as the monolayer capacity, the Langmuir constant, and the BET constant; see Appendix A for details) signify when these models are applied to the systems that break their original assumptions. At the same time, what makes an isotherm type different from another cannot be clarified when different isotherm models are used for different isotherm types. Building on the success of our statistical thermodynamic ABC^28,30^ and cooperative^36,37^ isotherms in modeling experimental sorption data with mechanistic insights, the objectives of our papers are

(i) to establish a consistent approach to model all isotherm types, attributing the difference to the sorbate–sorbate and sorbate–surface interactions in a systematic manner;
(ii) to generalize the language of the traditional sorption models (such as the monolayer capacity and the BET constant, see Appendix A) to the model-free concepts of partitioning and association coefficients that can be applied across the isotherm types;
(iii) to eliminate the apparent contradictions caused by applying the site-specific models alongside with cross-sectional area of sorbates for the purpose of surface area determination.

While our focus is chiefly on Types I–III, its natural connection to Types IV–VI will be established in reference to our recent work.^36,37^ (Note that we focus on the multi-stepwise Type VI-like sorption on heterogeneous materials,^37^ instead of the strict definition of “layer-by-layer adsorption on a highly uniform nonporous surface.”^6^) A comparison between the isotherm models and statistical thermodynamics will reveal and identify the stumbling block of the traditional approaches: inconsistent treatment of attractive and repulsive interactions which has confused the interpretation of biomolecular solvation and solubilization in the recent past.^31−35^ We will demonstrate how clarity is attained by treating attraction and repulsion on an equal footing.

## Theory

### Theoretical Foundation

#### Fluctuation Sorption Theory

Based on a rigorous statistical thermodynamic theory, we have shown that sorbate–sorbate and sorbate–interface interactions can be quantified from isotherm’s shape alone with the help of statistical thermodynamics.^27−30^ Here, we summarize our recent results using the statistical thermodynamic notation and the theoretical foundation (see Appendix A).^27,28,30^ The sorbate–sorbate interaction is quantified via the sorbate excess number, *N*_22_, and can be evaluated directly from the sorbate activity *a*_2_ dependence of the amount of sorption ⟨*n*_2_⟩, via
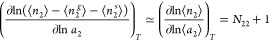
1which applies universally to any isotherm.^27,28,30^ (Note that ⟨*n*_2_^*s*^⟩ and ⟨*n*_2_^*g*^⟩, the number of sorbates in the solid and vapor reference states within the same interface (with the volume *v*), are much smaller than ⟨*n*_2_⟩, hence can be neglected.) The sorbate–interface interaction can also be calculated straightway from an isotherm via the sorbate–surface Kirkwood–Buff integral (KBI), as
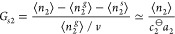
2where *c*_2_^⊖^ is the concentration of the saturated vapor, which comes from the definition of sorbate activity, and *a*_2_ = (⟨*n*_2_^*g*^⟩/*v*)/*c*_2_^⊖^.^28,30^*N*_22_ can also be related to its KBI counterpart, *G*_22_, as shown in Appendix A. The approximate forms in eqs 1 and 2, that are used in practice, are valid under the dominance of the amount of sorption in the surface excess, as has been justified for common isotherms.^30^

#### ABC Isotherm

Now we introduce a statistical thermodynamic isotherm derived from eq 1, referred to as the ABC isotherm,^28,30^ as the theoretical foundation for the present paper. We start by rewriting eq 1 in terms of the sorbate–sorbate KBI, as^28,30^
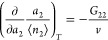
3awhich is integrated to yield^28,30^
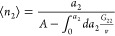
3bFrom eq 3b, a general isotherm was derived,^28,30^ based on a simple expansion of *G*_22_/*v*, i.e.,
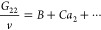
4awhere *A* and *B* represent the surface–sorbate interaction and the sorbate pair interaction at the interface, respectively (Appendix A). The interpretation of *C* as the sorbate triplet interaction at the interface is derived in Appendix B and explained in the next subsection. We emphasize here that the presence of the interface affects the sorbate pair and triplet interactions. Combining eqs 3b and 4a, a sorption isotherm (called the ABC isotherm) was derived^28,30^ as
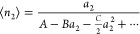
4b

The general quadratic function in the denominator shows that that eq 4b contains not only the BET and GAB models but also the Langmuir (*C* = 0, *B* < 0) as its special cases^28,30^ without their assumed site-specific and layer-by-layer adsorption mechanisms.^27,28,30^

### Isotherm Types as a Gradation of Sorbate–Sorbate Interaction

#### Absence of Sorbate–Sorbate Interaction Leads to the Linear (Type 0) Isotherm

**Figure 1:**
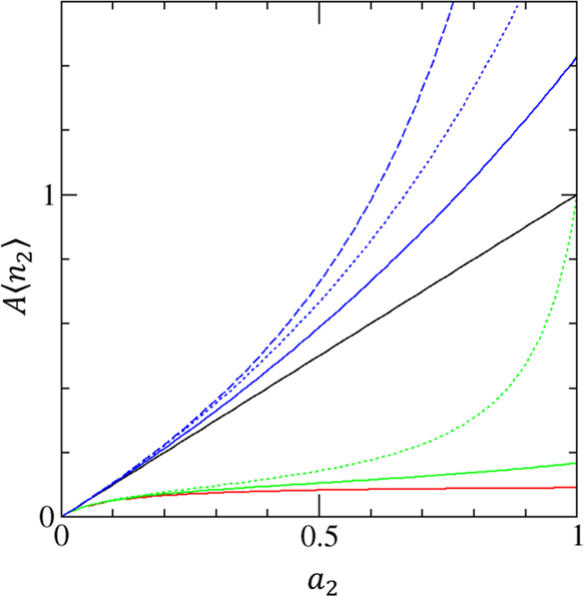
Statistical thermodynamic ABC isotherm can fit Types 0, I, II, and III single-handedly, which has been demonstrated by the normalized ABC isotherm, . Type 0 corresponds to  (black line). Type I comes from ,  (Table 1), exemplified by  (red line). Type II corresponds to  and  (Table 1); here keeping ,  (green solid line) and  (green dotted line) have been presented. Type III can be observed for  and  (Table 1), whose examples are plotted here at  (blue solid line), (0.5, 0) (blue dotted line), and (0.5, 0.5) (blue dashed line).

In this section, we demonstrate how our new view of sorption leads to a systematic classification of isotherms based directly on sorbate–sorbate and sorbate-sorbent interactions. We start with the linear or Type 0 isotherm. When sorbate–sorbate interaction is zero (*B* = *C* = 0), the ABC isotherm (eq 4b) reduces to ⟨*n*_2_⟩ = *a*_2_/*A* (Table 1 and Figure 1). This is consistent with the classical equation-of-states (EOS)-based approaches; adopting the ideal gas EOS interaction has led to the linear isotherm.^22,38−41^ Such a conclusion may appear in apparent contradiction to the site-specific adsorption models (Langmuir, BET, and GAB) that have been claimed to contain “no lateral interaction between adsorbed molecules”.^42−45^ Note, in the context of these models, “interaction” is synonymous with attraction. Indeed, in Type 0, the interactions among sorbate molecules are negligible both for the attractive and repulsive components. The absence of attractive interactions alone does not lead to *B* = 0; the repulsive interaction should also be negligible. Note that sorbate-sorbate interactions, by definition, are mediated by their interaction with the surface.

**Table 1:** Signs of the Parameters in the ABC Isotherm (Equation 4b) for Types 0, I, II, and III

type	A	B	C
0	+	0	0
I	+	–	0
II	+	–	+
III	+	+	+,0

#### Sorbate Pairwise Repulsion Underlies Isotherm Types I and II

Type I is characterized by *B* < 0, *C* = 0, which can be distinguished from Type II (*C* > 0) (Table 1 and Figure 1). This means that *G*_22_/*v* stays unchanged at its negative limiting value (i.e., *G*_22_/*v* at *a*_2_ → 0) independent of *a*_2_, i.e., the value for the sorbate pair in isolation in the proximity of the interface. Consequently, its constancy means that the sorbate–sorbate pairwise exclusion is not affected by the presence of surrounding sorbate molecules. Such an interpretation contrasts with the site-specific adsorption models for which “interaction” is synonymous with attraction.

What is the difference in the underlying molecular interaction between Type I and Type II? This question cannot be answered by the site-specific adsorption models themselves, such as the Langmuir, BET, and GAB. Indeed distinguishing monolayer adsorption from multilayer mechanism is difficult in the framework of these models because the Langmuir model cannot be derived as a special case of BET or GAB.^30^

This inability of the model-based approach contrasts with the clarity and ease afforded by our theory once a clear interpretation of *C* has been given. In our previous paper, *C* was interpreted merely as the difference between sorbate triplet and pair interactions.^28^ Here, a clearer interpretation of *C* is presented (see Appendix B) as
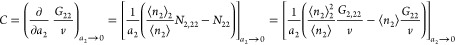
5Consequently, *C* represents how the sorbate–sorbate pairwise interaction in the presence of an extra probe sorbate (⟨*n*_2_⟩_2_*N*_2,22_/⟨*n*_2_⟩) changes from its absence (*N*_22_). A positive *C* (which is commonly encountered for Types II and III) represents the increase of sorbate–sorbate interaction caused by the presence of a third sorbate.

It is well known that the Type I behavior, analyzed routinely by the Langmuir model, is not limited to site-specific adsorption on a planar surface;^6^ Type I behavior has been observed for micropores that cannot be considered site-specific.^6,28,30^ In such a case, the constancy of *G*_22_/*v* can be achieved by confining the sorbates into separate pores so that only up to pairwise interaction is present between sorbates. In this context, −*G*_22_ signifies the volume occupiable per sorbate at the interface.

Thus, statistical thermodynamics has clarified that sorbate–sorbate exclusion, independent of the presence of another sorbate, is the basis for Type I behavior that distinguishes it from Type II. Such an independence can be realized by site-specificity assumed by the Langmuir model yet is not the exclusive mechanism.

### Sorbate Pairwise Repulsion Is Implicit Even in the Site-Specific Models

The signature of Type II behavior is *B* < 0, *C* > 0 (Table 1 and Figure 1), sorbate–sorbate interaction is repulsive (*B* < 0) yet becomes less so with increasing *a*_2_ (*C* > 0). This statistical thermodynamic view is in apparent contradiction with the traditional view (cf. BET and GAB) that assumes the lack of lateral sorbate interactions within an adsorption layer.^42,43^ How can we reconcile the apparent contradiction? To answer this question, here we show statistical thermodynamically that site-specific adsorption models contain sorbate pairwise exclusion implicitly, despite their claim otherwise.

For simplicity, let us take the Langmuir model as an example. Its statistical thermodynamic re-derivation of the Langmuir model is founded on the assumption that “[t]he adsorbed states belonging to any one surface atom are assumed to be independent of whether surrounding surface atoms are holding adsorbed molecules or not”.^46^ Expressing the adsorption isotherm on the surface, comprising *n*_*m*_ statistically independent adsorption sites with single maximum occupancy, each with the binding constant *K*_*L*_, the Langmuir model can be expressed as^46^
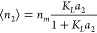
6aIn the Langmuir model, *n*_*m*_ is called the monolayer capacity. Using eq 6a in combination with eq 3a, we obtain
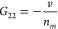
6b*G*_22_ has a dimension of volume, and when it is negative, −*G*_22_ can be considered as the “volume” occupied by a single sorbate molecule. It should be noted that a “molecular volume” cannot be defined unambiguously due to the cloud-like nature of the electron distribution within a molecule and is a concept introduced to assist an intuitive understanding of intermolecular arrangements. *G*_22_ reflects all of the effects of intermolecular interactions, both repulsive and attractive, and it is negative when the repulsive contribution is dominant. The excluded volume effect, which refers to the prohibition of the overlapping of molecules, is a major part of repulsive interaction. Accordingly, interpreting −*G*_22_ as the volume is justified only when *G*_22_ < 0. (This point will be elaborated further in the Results and Discussion section using eq 14.)

Thus, eq 6b has revealed that sorbate–sorbate exclusion is at work even for the Langmuir model which has claimed to contain no sorbate–sorbate interaction. Indeed, the volume per site on the right-hand side of eq 6b is equivalent to sorbate co-volume. This conclusion can also be reached by explicitly considering the statistical independence of site-specific adsorption (Appendix C).

### Langmuir Model versus the Gurvitsch Rule

The Langmuir model exhibits saturating sorption capacity at large *a*_2_. Similar saturating behavior is observed also in porous adsorbents, on which adsorbates have been considered to exhibit liquid-like density at high *a*_2_.^19,47,48^ This frequent observation, commonly referred to as the Gurvitsch rule,^19,47,48^ can be translated into the language of statistical thermodynamics as the similarity in value between the sorbate–sorbate KBI, *G*_22_, and its liquid-state counterpart, *G*_22_^(liq)^ (which is related to its molar volume, *V*_2_^(liq)^), as
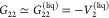
7Note that eq 7 is for the pure liquid of species 2 and a negligibly small contribution from isothermal compressibility has been omitted.^31,49^ We emphasize here that “the degree of molecular packing in small pores is affected by the pore size and shape”,^5^ which may make sorbate–sorbate *G*_22_ deviate from eq 7.

Thus, we have reached two different mechanisms that lead to the amount of sorption exhibiting saturation: Gurvitsch’s rule (eq 7) via sorbate packing versus the full coverage of adsorption sites in the Langmuir model (eqs 6a and 6b). How, then, can we distinguish the two mechanisms? In the case of the Langmuir model, we have shown that its underlying constancy of *G*_22_/*v* comes from sorbate–sorbate interaction unaffected by the presence of a third probe sorbate. In addition, the Langmuir model’s site-specific adsorption mechanism means that the sorbate–sorbate *G*_22_ is determined purely by adsorption site distribution. Thus, the signature of site-specific adsorption is a constancy of *G*_22_/*v* over all *a*_2_.

**Figure 2:**
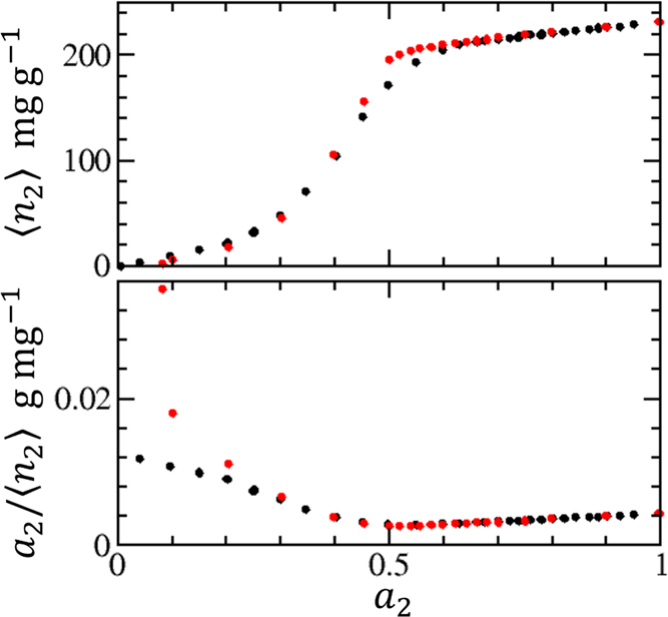
Revealing the non-site-specific nature of saturating isotherms. (a) Adsorption (black) and desorption (red) branches of water sorption on a pitch-based (PIT) hydrophobic activated carbon fibers with the slit size of 0.6 nm that have been measured by Nakamura et al.^50^ (b) Plot of *a*_2_/⟨*n*_2_⟩, whose gradient yields  via eq 3a. Even though a linear behavior above *a*_2_ > 0.5 shows the constancy of sorbate–sorbate exclusion that is observed also for site-specific adsorption, a negative gradient at lower *a*_2_ indicates sorbate–sorbate attraction characteristic of cooperative sorption and its negativity is not evident beyond *a*_2_ ∼ 0.5. Such a behavior is different from the constant  over all *a*_2_ expected for a site-specific sorption mechanism.

Here we take the adsorption of water on a pitch-based hydrophobic activated carbon (Figure 2)^50^ and examine whether its saturation in the amount of sorption comes from site-specific sorption. A plot of *G*_22_/*v* changes with *a*_2_, whose values are clearly different from the limiting value, showing that the site-specific mechanism is unlikely. Indeed, a positive *G*_22_/*v* below *a*_2_ ≃ 0.5, evident from the negative gradient of *a*_2_/⟨*n*_2_⟩ (Figure 2), is characteristic of sorption cooperativity. Thus, we have demonstrated the importance of *G*_22_ in identifying the sorption mechanism.

### Sorbate–Sorbate Attraction Underlies Type III

Type III behavior is characterized by a positive *B* and non-negative*C* (Table 1 and Figure 1). A sorbate, already present at the interface, attracts more sorbate molecules from the vapor phase through a favorable sorbate–sorbate interaction, even when the initial surface–sorbate interaction (*A*^–1^) is weak. As a result, *G*_*s*2_, which is proportional to ⟨*n*_2_⟩/*a*_2_ (eqs 2 and 4b), increases with *a*_2_. This mechanism is consistent with the IUPAC views on sorbate clustering present in Type III^6^ which has been captured statistical thermodynamically by the positive sign of *G*_22_.

The site-specific models, despite their ability to fit Type III, nevertheless suffers from a contradiction; it is difficult to reconcile sorbate cluster formation^6^ with the presumed absence of lateral sorbate–sorbate interaction.^42,43^ In addition, the presumed layer-by-layer adsorption mechanism is at odds with a small BET constant, *C*_*B*_(<2), required for the Type III behavior;^42,43^ when *C*_*B*_ is small, multilayer adsorption is more favorable than monolayer adsorption, which energetically prohibits the completion of monolayer. Thus, despite successful fitting, it is difficult to reconcile the Type III behavior with the basic assumptions of the site-specific models.

In contrast, according to our statistical thermodynamic framework, Type III differs from Types I and II only by the sign of *B*; deriving a separate isotherm model applicable only to Type III, such as the anti-Langmuir model,^51,52^ has been made redundant. (Note that the anti-Langmuir model corresponds to *B* > 0 and *C* = 0.) Thus, our ABC isotherm (eq 4b) is capable of modeling Types I–III solely without any contradictions or model assumptions.

**Figure 3:**
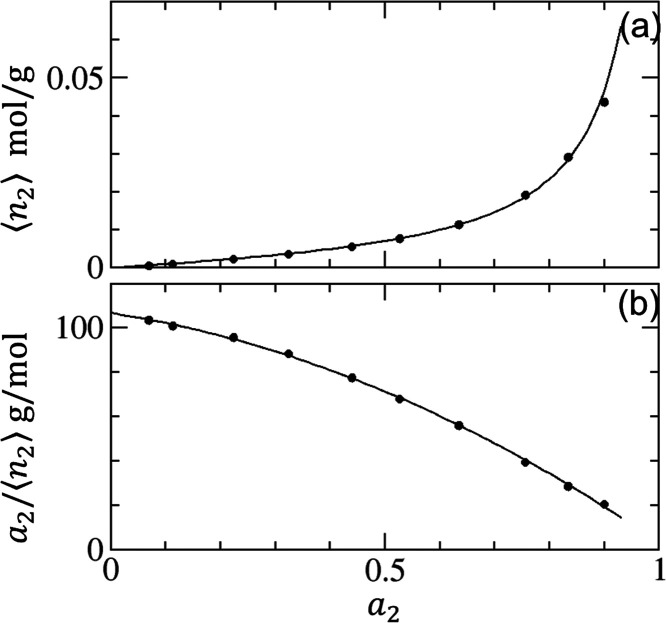
(a) Sorption isotherm of water on a green coconut pulp at 30 °C measured by Lavoyer et al. (circles),^54^ presented with the fitting by the statistical thermodynamic ABC isotherm (eq 4b) carried out in (b). (b) Determination of the parameters *A*, *B*, and *C* of the ABC isotherm (eq 4b) using the reciprocal form of eq 4b, *a*_2_/⟨*n*_2_⟩ = *A* – *Ba*_2_ – (*C*/2)*a*_2_^2^, with the nonlinear regression results (with the units of g/mol) of *A* = 106.7, *B* = 38.04, *C* = 130.9. The positive *B* signifies sorbate–sorbate attraction and the positive *C* shows its cooperative strengthening according to eq 5.

Our focus on the gradation of sorbate–sorbate interaction, instead of site-specific adsorption, rationalizes why Type III behavior is seen in disparate classes of materials,^3,25,53,54^ such as “nonporous or macroporous surfaces which interact very weakly with adsorbate molecules”^53^ and “[f]oods that are rich in soluble compounds such as sugars”.^54^ It is hard to imagine that the latter can be modeled by site-specific, layer-by-layer adsorption on planar interfaces. Yet, the ABC isotherm (eq 4b), being free of such restrictive model assumptions, is applicable to food with soluble components (Figure 3). Sorbent, when dissolved at the interface, can enhance the clustering of sorbates around it, thereby strengthening sorbent–sorbate interaction even though it is weak at *a*_2_ → 0. This is underscored by the positive *B* signifying sorbate–sorbate attraction and the positive *C* showing its cooperative strengthening according to eq 5 (Figure 3). Moreover, interfacial and solution-phase KBIs obey an analogous relationship; hence, the presence of the soluble component does not pose any difficulty for our theory (Appendix D). This is the underlying mechanism for favorable sorbate–sorbate interaction in foods that leads to a Type III behavior that can be captured without any difficulties by our ABC isotherm.

Thus, the Type III behavior is observed when sorbate–sorbate interaction is attractive in general. The wide applicability of our theory (i.e., both adsorption and absorption with any interfacial geometry or porosity even with sorbent dissolution^28^) has eliminated the need for force-applying the site-specific models to a system that breaks their basic assumptions.

### Limitations of the EOS Approach to Isotherms

The site-specific models have been applied routinely to the systems without site-specific adsorption, despite a long history of questioning such an approach.^22^ The dominance of the site-specific models, in our view, has been perpetuated by the failure of the alternative approach based on the equation of states (EOS) for the spreading pressure (Π), to derive the Langmuir, BET, and GAB isotherms. The foundation of the EOS-based approach is the relationship between the amount of sorption ⟨*n*_2_⟩ and the spreading pressure, Π, via^1,27^
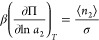
8awhere *β = 1/RT* and σ is the surface area of the interface, arising, in our view, from the restrictive requirement for the planar nature of the interface of the classical Gibbs isotherm. Note that the introduction of an EOS for Π implicitly assumes the “surface phase” (denoted as the superscript *s*) as being separate from the vapor phase (denoted as *v*). This necessitates an equilibrium condition between the two as
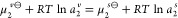
8bfrom which the sorbate activity in the surface phase is related to that of the vapor phase as
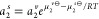
8cHowever, introducing the surface phase will complicate the derivation of isotherms, as we will demonstrate below. First, if we take the standard approach via the surface-vapor equilibrium condition (eq 8b), it makes the surface phase activity, *a*_2_^*s*^, different from the vapor phase activity *a*_2_^*v*^ (≡*a*_2_). While the vapor phase activity is the common variable for isotherms, the surface phase activity *a*_2_^*S*^, if chosen for *a*_2_ in eq 8a, needs to be calculated using the equilibrium condition (eq 8c), that requires additional pieces of information, μ_2_^*v*⊖^ and μ_2_^*s*⊖^, which may involve additional cumbersome work. This necessitates a more tractable approach based on an EOS assumed for the surface phase, expressing Π in eq 8a as a function of ⟨*n*_2_⟩/σ, as the two-dimensional equivalent for sorbate density. This approach, however, suffers from complications:⟨*n*_2_⟩/σ appears on both sides of eq 8a, whereas there is only one *a*_2_. Consequently, the resultant isotherm from eq 8a with an EOS usually takes the form of *a*_2_ as a function of ⟨*n*_2_⟩/σ,^22,38,41^ instead of a more common form of ⟨*n*_2_⟩ as a function of *a*_2_.

This explains why the EOS approach has failed to rederive the site-specific models, such as the Langmuir, BET, and the GAB. Incorporating the sorbate excluded volume in EOS has led to the Volmer model,^41^ for which the apparent affinity constant decreases with relative pressure in contrast to the constancy of the Langmuir constant;^1,39^ the EOS corresponding to the Langmuir model, the simplest of the site-specific models, has nevertheless been shown to have a complicated mathematical form.^39^ Adopting van der Waals EOS has led to the Hill-de Boer model,^22,38^ which is more complex in form than the BET and GAB models. Thus, the gap between the site-specific and EOS models has remained unfilled for decades. (Note that the EOS-based isotherms can, in principle, be linked to the excess number and KBIs via eqs 1, 2, and 3a).

In contrast, our approach is based on expanding sorbate–sorbate KBI. The key was to keep *a*_2_ as a single variable. This has led to the ABC isotherm as a site-free generalization of the Langmuir, BET, and GAB models.

## Results and Discussion

### Universal versus Model-Specific Descriptors

The analysis and modeling of Types I–III isotherms have been dominated by site-specific models (e.g., Langmuir, BET, and GAB) despite the nonspecific nature of many isotherms.^22^ As a result, the monolayer capacity and the BET constant have shaped the thinking of adsorption scientists for generations. Therefore, we must explore how these traditional concepts can still be used yet with a renewed statistical thermodynamic interpretation that replaces the unrealistic language of the site-specific models.

Our strategy for achieving this objective is through a statistical thermodynamic generalization of the site-specific models, by taking advantage of their correspondence with the ABC isotherm. In this way, not only can the basic features of the six IUPAC types be captured based on their underlying interactions but also the root cause of confusion (i.e., the need for force-fitting site-specific models to non-site-specific isotherms as discussed in the Introductionsection) can be eliminated.

Before presenting our main results, let us briefly summarize what we have tentatively achieved toward achieving this objective in our recent papers. Let us start from the correspondence between the “AB isotherm” (i.e., eq 4b with *C* = 0) and the Langmuir model (eq 6a). For the Langmuir model, the amount of sorption approaches the monolayer capacity, *n*_*m*_, at *a*_2_ → 1. We emphasize that the monolayer capacity is given *a priori* by the Langmuir model, without any further explanation of its origin. In contrast, the amount of sorption in the AB isotherm tends at *a*_2_ → 1 to the interfacial capacity, *n_I_*, defined as
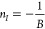
9awhen *B* is larger in magnitude than *A*. The advantage of eq 9a over the Langmuir model is the availability of its microscopic interpretation (via *B*, see eq A6 in Appendix A) in terms of the sorbate–sorbate KBI and the interfacial volume as
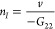
9bThus, according to eqs 9a and 9b, the amount of sorption saturates at *n_I_*, which is determined solely by the sorbate–sorbate interaction at the interface at the *a*_2_ → 0 limit. This can be appreciated by the molecular interpretation of *C* = 0 in eq 5, that sorbate–sorbate interaction is not affected by the presence of the third sorbate. Hence, *G*_22_/*v*, according to eq 4a, remains unchanged at the value at the *a*_2_ → 0 limit, which characterizes the isotherm throughout *a*_2_.

Moreover, eq 9b marks a departure from the site-specific models; −*G*_22_ signifies the sorbate co-volume, i.e., the volume around a probe sorbate into which other sorbate molecules cannot penetrate. Since sorbate co-volume is the measure of volume that a sorbate occupies, *v*/(−*G*_22_) counts the number of sorbates occupiable at the interface.

Thus, we have clarified the signature of Type I isotherms statistical thermodynamically without relying on the site-specific Langmuir model: sorbate–sorbate exclusion remains unchanged at its limiting value at *a*_2_ → 0.

### Interface/Vapor Partition Coefficient Replaces the Langmuir and BET Constants

In our previous paper, we have already derived, via a comparison of the ABC isotherm with the BET and GAB models, the statistical thermodynamic generalization of the Langmuir and BET constants, which has the following form^28^
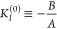
10awhere the superscript (0) emphasizes that this quantity is defined at the *a*_2_ → 0 limit. The goal of this section is to attribute a clear meaning to (and hence an appropriate name for) *K*_*I*_^(0)^. To do so, let us start by expressing *K*_*I*_^(0)^ statistical thermodynamically in terms of the KBIs, as
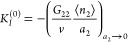
10bUsing the interfacial capacity, *n_I_* (eq 9b), eq 10b can be rewritten as
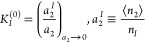
10c

Through eq 10c, *K*_*I*_^(0)^ has acquired a new interpretation as the interface/vapor partition coefficient. This is based on considering *a*_2_^*I*^ as the interfacial sorbate activity. Since *a*_2_^*I*^ = ⟨*n*_2_⟩/*n*_*I*_ is analogous to *a*_2_ = *c*_2_/*c*_2_^⊖^ in the vapor phase (i.e., the sorbate concentration relative to that of saturated vapor at *a*_2_ → 1), *a*_2_^*I*^ can be interpreted as the amount of sorption relative to *n*_*I*_ (i.e., the sorption capacity as the limiting amount of sorption at *a*_2_ → 1 under *C* = 0). Alternatively, *a*_2_^*I*^ can be interpreted more intuitively via eqs 10c and A3, as
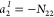
11The positive *a*_2_^*I*^ comes from the negative *N*_22_, reflecting sorbate–sorbate exclusion as the signature of Type I. (Note, unlike the constant *G*_22_/*v*, that *N*_22_ increases with *a*_2_ because ⟨*n*_2_⟩ in *N*_22_ = ⟨*n*_2_⟩*G*_22_/*v* increases with *a*_2_.) A larger sorbate–sorbate deficit number, according to eq 11, leads to a larger sorbate activity. This makes intuitive sense because sorbate activity, or relative vapor pressure, is higher with a stronger sorbate–sorbate repulsion.

Thus, the statistical thermodynamic generalization of the Langmuir and BET constants, *K*_*I*_^(0)^, has acquired a clear interpretation via statistical thermodynamics as the interface/vapor partition coefficient. (This is reminiscent of the previous attempts to relate the Kirkwood–Buff approach to the hydration shell/bulk partition coefficient^55^ defined under hydration shell model and accessible surface area.^56^) This interpretive clarity contrasts with the current understanding of the BET constant summarized in the IUPAC report: “[a]ccording to the BET theory, [the BET constant] is exponentially related to the energy of monolayer adsorption.”^6^ In this context, *K*_*I*_^(0)^ is exponentially related to the free energy of sorption; whether this free energy is dominated by the energy or entropy can be revealed by the temperature dependence of an isotherm.^57^

Thus, we have shown that *K*_*I*_^(0)^ (the statistical thermodynamic generalization of the Langmuir and the BET constants) and *a*_2_^*I*^ have a direct and model-free link to sorbate distribution at the interface. The interface/vapor partition coefficient introduced at the *a*_2_ → 0 limit can straightforwardly be generalized to finite sorbate activity (Appendix E). When *B* > 0, the above formalism does not apply but can be interpreted as an infinite series of sorption processes, reminiscent of indefinite self-association model (see Appendix F).^58−62^ This generalization has been made possible by a close analogy between solution and interface as the foundation of the ABC isotherm (Appendix D).

### Type-Specific Descriptors for All Six Isotherm Types

The vapor/interface partition coefficient *K*_*I*_^(0)^ is the statistical thermodynamic generalization of the Langmuir and BET constants and is applicable to Types I and II regardless of the adsorption specificity. In these types, −*RT* ln *K*_*I*_^(0)^ can be interpreted as the transfer free energy of a sorbate from vapor to the interface. This is the generalization of the “surface energy” in the surface characterization literature defined as +*RT* ln *K*_*L*_ (the commonly adopted positive sign signifies the desorption process) via the Langmuir constant, *K*_*L*_.^63−66^

The vapor-to-interface transfer energy has a clear relevance to the descriptor for Types IV–VI, −*RT* ln *A*_*m*_, signifying the free energy of sorbing *m* sorbate molecules cooperatively from the vapor phase (Appendix F). The descriptor for Type III is analogous to the binding constant in the infinite series of binding (Appendix F), reflecting how a sorbed molecule brings in more sorbates. Note that the forced application of the BET and GAB models to Type III is oblivious to the need for a descriptor different from the one for Types I and II.

Thus, in addition to the universal descriptors of interactions (*G*_*s*2_, *G*_22_, and *N*_22_), our theory offers the three model-independent processes (each with an equilibrium constant and free energy) that can characterize all isotherms of the six Types: the vapor-to-interface transfer for Types I and II, sorbate–sorbate association for Type III, and cooperative sorption for Types IV–VI.

### Overcoming the Difficulties Caused by the Site-Specific Models

#### Surface Area Overestimation for Porous Materials

Since the BET model is a restricted case of the ABC isotherm, fitting the ABC isotherm to experimental data is much easier than the BET model.^30^ Nevertheless, a common practice is force-adapting the BET model to isotherm data, which leads to systematic inaccuracies in surface area estimation, especially for porous materials. How such inaccuracies arise can be demonstrated by force-constructing a BET plot from the ABC isotherm (eq 4b), as
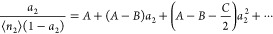
12Only under *C* = 2(*A* – *B*) can the right-hand side of eq 12 become a linear function of *a*_2_, known as the linear BET plot.^30^ However, when this condition is not satisfied, the BET plot becomes nonlinear. This affects the gradient of the BET plot, as can be seen by differentiating eq 12 with respect to *a*_2_ as
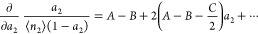
13For porous materials, the isotherm is cooperative, which is characterized by a large positive *C*. When *C*/2 > *A* – *B*, the gradient of the BET plot decreases with *a*_2_. If a “linear region” were to be identified, a negative  contributes to reducing the gradient of the “BET plot”, which is interpreted as (*C*_*B*_ – 1)/*C*_*B*_*n*_*m*_ ≃ 1/*n*_*m*_. (Note that this approximation is justified under a sufficiently large *C*_*B*_ necessary for a valid surface area determination). This leads to an underestimation of 1/*n*_*m*_; hence, the overestimation of *n*_*m*_ and therefore the BET surface area is overestimated. This trend is consistent with the recent papers that have reported the tendency for the BET model to overestimate the specific surface area for pores larger than about 1 nm.^67,68^ Thus, force linearization of eq 12, necessitated by the site-specific BET model, leads to systematic inaccuracies in surface area estimation.

#### Cross-Sectional Areas as Sorbate–Sorbate Exclusion at the Interface

Indispensable to surface area estimation are the cross-sectional areas of probe sorbates. A combined use of the cross-sectional areas and the site-specific adsorption models has given rise to conceptual difficulties; while the cross-sectional areas inherently assume “the liquid form of close-packed structure”^69^ for which repulsive interactions play an important role,^70,71^ the site-specific models have traditionally assumed the lack of lateral interactions. Having clarified the role of sorbate–sorbate repulsion even in Type II, we are now in the position of reconciling the contradictory perspectives of the past.

How cross-sectional areas should be evaluated has been the subject of ongoing debate.^19,69,72−78^ Recent molecular simulations^76−78^ have transformed the debate away from the old approaches (e.g., “Molecular models were built for each compound and a shadowgraph was taken with a point light source 6 feet away”^74^) to a statistical elucidation of interfacial structure. Therefore, it is timely to redefine the cross-sectional area in a statistical thermodynamic manner and to show how it is embedded consistently in the theory of sorption. For gases and solutions, the excluded volume of a molecule labeled as species 2, *b*_2_, is defined in terms of the radial distribution function, *g*_22_, as
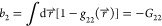
14which is related to KBI. When a pair of van der Waals molecules, which can modeled classically using *U*_22_(*r⃗*) as the potential energy, is in isolation, *b*_2_ can be evaluated using *g*_22_(*r⃗*) = exp(−*U*_22_(*r⃗*)/*RT*), from which *b*_2_ is close in value to the van der Waals co-volume. Equation 14 does not need any alterations when applied to sorbates at the interface, with the only condition that sorbate–sorbate distribution, *g*_22_, is conditional, subject to the presence of the interface, unlike the case of the isolated pair. (Such a conditional nature of sorbate–sorbate interaction at the interface was observed for the simulated hydrophobic association near self-assembled monolayer of surfactants.^79^)

The cross-sectional area, σ_2_, can be calculated straightway from *b*_2_ by assuming a spherical shape. Previously, *b*_2_ or σ_2_ were the parameters defined outside of the BET model yet were used in conjunction with the BET model. In contrast, our statistical thermodynamic theory incorporates *b*_2_ or σ_2_ within *N*_22_, as
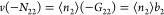
15aor assuming a two-dimensional system, eq 15a can be rewritten for the two-dimensional KBI and the interfacial surface area σ as
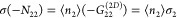
15bHere, the cross-sectional area, σ_2_, enters as the two-dimensional sorbate–sorbate KBI.

With this setup, here we introduce our recent approach to specific surface area estimation.^30^ In contrast to the ambiguity with which the “completion of monolayer” in the BET model has been defined and probed, we have employed the sorbate deficit number, −*N*_22_.^30^ The activity at which the deficit number takes a maximum (referred to as Point *M*) is the statistical thermodynamic definition of interfacial coverage.^30^ Consequently, eq 15b should be applied at Point *M*, as
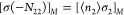
16What we evaluate in eq 16, namely, [σ(−*N*_22_)]_*M*_, indeed signifies the area covered by sorbates. This can be understood in the following manner: σ is the total area of the interface, and −*N*_22_ signifies the occupancy ratio because
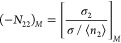
17is the cross-sectional area-to-area per sorbate ratio. The occupancy ratio becomes 1 when the surface is fully covered by sorbates.

Recently, the cross-sectional area’s strong dependence on *a*_2_ has been reported from the simulation of pores,^76−78^ which reflects the fact that σ_2_ derives from *g*_22_ at the interface, in a manner dependent on sorbate concentration thereat. This is consistent with the statistical thermodynamic picture that estimating σ_2_ requires the quantification of sorbate–sorbate interaction at a particular interface. Thus, what we have proposed here is the need for expressing all of the factors involved in surface area estimation statistical thermodynamically in terms of sorbate–sorbate distribution at the interface.

## Conclusions

This paper has aimed to rectify the unsatisfactory state of the art of sorption isotherm analysis, namely, (i) multiple isotherm models, each assuming a different sorption mechanism, being able to fit the same experimental data equally well, thereby providing no conclusive insights; (ii) routine application of the popular site-specific isotherm models (e.g., Langmuir, BET, and GAB) to the systems that break their basic assumptions. Our strategy was not to construct yet another isotherm model but to start from the fundamentals of statistical thermodynamics based on a generalization of the Gibbs isotherm to arbitrary interfacial geometry and porosity.

Our chief focus was on IUPAC Types I–III. A single model-free isotherm (i.e., the ABC isotherm), founded directly on the statistical thermodynamic fluctuation theory, was able to capture Types 0 and I–III solely. The different Types emerge from the gradation of sorbate–sorbate attraction and repulsion (as summarized in Table 1, Figure 1). The interpretive difficulties and confusions of the site-specific models, arising from their preferential bias on attractive interactions while incorporating sorbate–sorbate repulsion only implicitly, have been overcome. In addition, how the systematic inaccuracies in surface area estimation arise from the force-adaptation of site-specific models has also been identified. Such historical difficulties and inaccuracies are not limited to the study of sorption alone; the same bias toward attractive interactions led to historical controversies in protein stabilization and denaturation, conformational changes, and small molecule solubilization.^31−35^

The current analysis of sorption has been shaped by the Langmuir, BET, and GAB models, despite their highly idealized (or even unrealistic) assumptions. Attempts to quantify effects using Langmuir, BET, and GAB models have produced parameters (such as the BET constant and specific surface area) that themselves have led to confusion. Appreciating this reality, we have provided a new interpretation of the Langmuir and BET constants as the vapor/interface partition coefficient, *K*_*I*_. This new interpretation is based directly on statistical thermodynamic fluctuation theory. Adopting this new interpretation is advantageous not only because *K*_*I*_ is applicable beyond site-specific sorption and is free from the confusion arising from their force application but also offers a smooth connection to the binding constants that characterize Types III and IV–VI. Thus, the fewer quantities with universal applicability can replace 100+ models currently used,^7−13^ thereby the isotherm analysis can be decluttered. Yet, at the same time, the wealth of data fitting from historic papers can readily be reinterpreted in a statistical thermodynamic light because the Langmuir and BET constants have been given a new model-free interpretation.

In a forthcoming paper, we will extend our theory to solid/solution interface to generalize our isotherm equations to sorption from solution.^80−82^

## Appendix A: Notation, Foundation, and Isotherm Models

### Notation

Throughout this paper, we use the term “sorption” unless there is a need to distinguish adsorption, absorption, or desorption. We employ the statistical thermodynamic notation of our previous papers.^27−30,36,57^ The sorbent and sorbate are referred to as molecular species 1 and 2, and *n*_2_ and *a*_2_ are the number (of molecules) and activity of sorbates, respectively. The ensemble average is denoted by ⟨⟩; hence, the sorption isotherm is the dependence of ⟨*n*_2_⟩ on *a*_2_ at the temperature *T*. *R* designates the gas constant. The deviation of *n*_2_ from the mean is denoted by δ*n*_2_ = *n*_2_ – ⟨*n*_2_⟩, through which the number fluctuation is expressed as ⟨δ*n*_2_δ*n*_2_⟩. The correspondence to the IUPAC notation is given as *n* = ⟨*n*_2_⟩ and *p*/*p*_0_ = *a*_2_, where *p*_0_ is the pressure of the saturated vapor.

### Foundation

Our theory is applicable to any interfacial geometry and porosity, which was enabled by the generalization of the Gibbs isotherm^27^ under the universally justifiable postulate on a finite-ranged nature of an interface.^27,28,30,57^

The objective of the fluctuation sorption theory is to quantify interactions using number correlations between species.^31,32,83−86^ The surface–sorbate interaction is quantified by the surface–sorbate Kirkwood–Buff integral (KBI), *G*_*s*2_, defined as^27,28,30^

A1

where *v* is the volume of the interface, and ⟨*n*_2_^*s*^⟩ and ⟨*n*_2_^*g*^⟩ are the number of sorbates in the solid and vapor reference states, respectively. (Note that *G*_*s*2_ is different from *G*_12_. *G*_*s*2_ quantifies the net distribution of sorbate molecules in the vicinity of the surface, whereas *G*_12_ represents the sorbent–sorbate Kirkwood–Buff interaction based on a pairwise distribution of sorbent and sorbate molecules.) The sorbate–sorbate interaction at the interface is defined as^27,28,30^

A2

*G*_22_ is related to the excess number of sorbates around a probe sorbate as in the following:^27,28,30^

A3

The advantage of using *G*_*s*2_, *G*_22_, and *N*_22_ is in their direct link to sorbate distribution at the interface. *G*_*s*2_ and *G*_22_ can be expressed in terms of the sorbate–surface and sorbate–sorbate distribution functions, *g*_*s*2_ and *g*_22_, as^27,28,30^

A4

A5

Consequently, the sorption mechanism underlying an isotherm can be clarified by quantifying surface–sorbate and sorbate–sorbate interactions via *G*_*s*2_, *G*_22_, and *N*_22_ that have a clear physical meaning.

The constants *A* and *B* in eq 3b have been attributed to the following statistical thermodynamic interpretation:^28^

A6

where *A* is related to the surface–sorbate KBI at *a*_2_ → 0 limit and *B* is the sorbate–sorbate KBI per interfacial volume at the same limit.^28,30^ A clear interpretation of *C* will be given in the Theory section and Appendix B of this paper.

### Isotherm Models

The Langmuir, BET, and GAB models are the most commonly used isotherms for Types I–III. The Langmuir model for Type I, with the Langmuir constant *K*_*L*_ and the monolayer capacity, *n*_*m*_, has the following form:^87^

A7

The BET model^18,19^ is an extension of the Langmuir model by introducing multilayer adsorption, with the BET constant, *C*_*B*_, being related exponentially to the energy of monolayer adsorption, which has the following form:

A8

The GAB model^20−22^ has extended the BET isotherm further by incorporating the GAB constant, *K*_*G*_, to account for the difference in binding between the first and outer layers, as

A9

These models, derived assuming the layer-to-layer adsorption mechanism, have been applied to model Types I–III isotherms.

In contrast, our model-free statistical thermodynamic approach to isotherms employs the ABC isotherm (eq 4b) and the cooperative isotherm (Appendix F).

## Appendix B: Sorbate Triplet Contribution to the ABC Isotherm

Here we derive a statistical thermodynamic interpretation of *C*. To do so, let us start from eq 4a, which leads to

B1

where *β = 1/RT* and *G*_22_ can be expressed in multiple, equivalent ways, as

B2

where ⟨⟩_2_ denotes the inhomogeneous ensemble average in the presence of 2; an “inhomogeneous ensemble” is defined here as a statistical ensemble of a system that contains a sorbate (species 2) at origin.^88,89^ According to the inhomogeneous solution theory, the average taken in this inhomogeneous ensemble (⟨⟩_2_) can be related to the one in the homogeneous ensemble (i.e., ⟨⟩) via^88,89^

B3

Equation B2 also shows an alternative expression via the variance, δ*n*_2_ = *n*_2_ – ⟨*n*_2_⟩.

Our goal is to provide a simple statistical thermodynamic interpretation of *C* in eq B1. To do so, combining eqs B1 and B2 involves the following differentiation:

B4

The evaluation of the first term of the right-hand side involves

B5

where, from eq B2,

B6a

In an analogous manner, *G*_2,22_ (sorbate–sorbate KBI in the presence of a sorbate) can be introduced as

B6b

Subtracting eq B6a from eq B6b yields

B7

where, in the final step of eqs B7, B2 was used. Using eq B2, the second term of eq B4 can be evaluated as

B8

Noting that *N*_22_ → 0 at the *a*_2_ → 0 limit, we combine all of the above in eqs B1 and B4, yielding

B9

which signifies the difference between sorbate–sorbate KBI in the presence and absence of a sorbate molecule as an external field source.

## Appendix C: Sorbate–Sorbate Distribution Function for a Site-Specific Binding Model

As shown in the main text, the traditional isotherm models focus on site-specific sorbate–surface interaction while neglecting sorbate–sorbate interaction between neighboring sites. Here, we provide an alternative derivation of eq 6b from a statistical perspective. Let ⟨*n*_2_⟩_2_ be the number of sorbates at the interface, conditional to the presence of the probe sorbate. When site-specific binding on a site is statistically independent of other sites, ⟨*n*_2_⟩_2_ is simply the total number of sorbates minus probe, hence

C1

Combining eq C1 with the definition of the excess number (eqs A2 and A3), rewritten using inhomogeneous ensemble (eq B3), we obtain

C2

Using the relationship between *N*_22_ and *G*_22_ (eq A3), we obtain

C3

Note that ⟨*n*_2_⟩ in this context is the number of binding sites.

## Appendix D: Solution-Surface Analogy Underlying the ABC Isotherm

The ABC isotherm, expressed as a polynomial expansion of *a*_2_/⟨*n*_2_⟩, offers a powerful analogy to the solution theory, helpful for treating solid and liquid sorbents in a unified manner. The solution-phase analogy to the ⟨*n*_2_⟩/*a*_2_ plot can be appreciated via a version of eq 3a,

D1a

compared with its liquid-state analogue using the molar concentration *c*_2_

D1b

where *G*_22_^(*N*_1_)^ is the KBI analogue for the constant *N*_1_ ensemble, defined as

D2

which will be linked to the standard KBI in μ_1_ ensemble.

To derive eq D1b, let us consider a semiopen system in the solution phase of volume *V*, open to species 2 but closed to species 1. Let the numbers of species *i* be denoted as *N*_*i*_. The application of semi-grand ensemble yields

D3

The right-hand side of eq D3 can be rewritten using eq D2, as

D4

Equation D4 can be transformed into a compact form as

D5

Multiplying both sides with *V* leads to eq D1b.

We have shown that the interface (eq D1a) and solution (eq D1b) obey the analogous equations when constant *N*_1_ ensembles are chosen. For solutions, it is common to employ the grand canonical ensemble to express fluctuations and KBIs. Transforming fluctuation from constant *N*_1_ to constant μ_1_ ensemble is carried out straightforwardly via statistical variable transformation under the invariance of mole ratio *N*_2_/*N*_1_ and its variance.^90,91^ The process of variable transformation was carried out in our previous paper (Equations 14–17 of ref (28)), which yields

D6

Thus, a comparison between sorption and solution theories leads to the interpretation of *a*_2_/*c*_2_ = γ_2_^(*c*)^ as the molarity-based activity coefficient^92^ is generalized to the interface.

## Appendix E: The Interface/Vapor Partition Coefficients at Finite *a*_2_

The interface/vapor partition coefficient introduced at the *a*_2_ → 0 limit can straightforwardly be generalized to any sorbate activity. This can be achieved by simply eliminating the *a*_2_ → 0 limit, and consequently the superscript (0) in eqs 2, 9b, 10a−10c, and 11, as

E1

This generalization leads directly to a graphical method of determining *K*_*I*_ directly from the isotherm data. A combination of it with eqs 2 and 3b leads to

E2

Equation E2 shows that a gradient of a plot of  against *a*_2_ gives *K*_*I*_. We must be careful here of the fact that the amount of sorption for the ABC isotherm can exceed *n*_*I*_; hence, interpreting ⟨*n*_2_⟩/*n*_*I*_ as activity breaks down when ⟨*n*_2_⟩ > *n*_*I*_. Yet *A*, *B*, and *C* are all defined at the *a*_2_ → 0 limit; hence, this interpretation is valid for low *a*_2_. Thus, we have successfully emancipated the Langmuir and BET constants from being restricted to a site-specific binding constant.

## Appendix F: Link to Types III–VI

First, we show that the vapor-to-interface transfer free energy of a sorbate has a natural link to the key quantity in cooperative sorption for Types IV–VI. We start with the statistical thermodynamic cooperative isotherm^36,37^

F1

where *m* is the number of sorbates cooperatively sorbed to a patch, *N*_*m*_ is the number of patches, and −*RT* ln *A*_ν_ calculated from *A*_ν_ is the free energy of moving ν sorbates from saturated vapor to the interface, respectively. We can easily see that eq F1 reduces to the Langmuir–Freundlich model under *A*_1_ = 0, as

F2

In the literature on surface energy characterization, *f*_C_ = −*RT* ln *A*_*m*_ has often been referred to as “adsorption energy”. Our −*RT* ln *A*_*ν*_ is its statistical thermodynamic generalization, which has a clear link to the vapor to interface transfer free energy.

Note that the cooperative isotherm (eq F1) was applied to the experimental water adsorption isotherms on hydrophobic activated fibers (with pore widths of 0.5 and 0.6 nm) and pitch resin-based activated carbon materials with varying slit sizes (0.5, 0.6, 1.0, and 1.1 nm).^36^ Moreover, heterogeneous materials with multiple pore sizes were modeled successfully by multiple terms of the cooperative isotherm. Such an approach was applied to experimental isotherm data, including NH_3_ adsorption on Kuf-1a (a hydrogen-bonded organic framework), water adsorption on an aluminophosphate molecular sieve, and CO_2_ adsorption on PCN-53 (a metal–organic framework), from which the sorbate cluster number and the free energy of sorption were determined for mechanistic insights.^37^

Second, we demonstrate that the AB isotherm can be interpreted in a manner relatable to Types I–II and IV–VI, even for the parameter range corresponding to Type III (*B* > 0 with *C = 0*). To do so, let us rewrite the AB isotherm as

F3

where *K*_*AL*_ = *B*/*A* will be referred to as the anti-Langmuir constant. Carrying out Maclaurin expansion leads to

F4

This means that −*RT* ln *A*^–1^ = *RT* ln *A* is the sorption free energy of the first sorbate, with the subsequent sorbates, each with the sorption free energy of −*RT* ln *K*_*AL*_. In this manner, eq F4 is reminiscent of the indefinite self-association model.^58−62^ Generalization of eq F4 to the ABC model will be straightforward yet less rewarding because of the complex expressions for the association constant at each step. Nevertheless, Type III isotherms can be interpreted as an infinite successive sorption process.
